# Mesenchymal Stem Cell-Derived Exosomes miR-143-3p Attenuates Diabetic Kidney Disease by Enhancing Podocyte Autophagy via Bcl-2/Beclin1 Pathway

**DOI:** 10.3390/biomedicines14010184

**Published:** 2026-01-14

**Authors:** Wenze Song, Jiao Wang, Lulu Guan, Yun Zou, Jiarong Liu, Wen Chen, Jixiong Xu, Wei Cai

**Affiliations:** 1 Department of Medical Genetics and Cell Biology, School of Basic Medical Sciences, Jiangxi Medical College, Nanchang University, Nanchang 330031, China; 2 Department of Endocrinology and Metabolism, The First Affiliated Hospital, Jiangxi Medical College, Nanchang University, Nanchang 330031, China; 3 Jiangxi Clinical Research Center for Endocrine and Metabolic Disease, Nanchang 330031, China; 4 Jiangxi Branch of National Clinical Research Center for Metabolic Disease, Nanchang 330031, China

**Keywords:** mesenchymal stem cells, exosome, diabetic kidney disease, podocyte, autophagy

## Abstract

**Objective**: Diabetic kidney disease (DKD) is characterized by podocyte injury and impaired autophagy. Bone marrow mesenchymal stem cell-derived exosomes (BMSC-Exos) exhibit therapeutic potential for DKD, yet their mechanisms remain unclear. This study investigated whether BMSC-Exos restore podocyte autophagy via the miR-143-3p/Bcl-2/Beclin1 axis to delay DKD progression. **Methods**: A high-glucose (HG)-induced podocyte injury model was established using mouse podocytes (MPC5). Autophagy-related proteins (Beclin1, Bcl-2, LC3) and the injury marker desmin were analyzed by Western blot and immunofluorescence (IF). High-throughput sequencing identified BMSC-Exos-enriched miRNAs, with the miR-143-3p/Bcl-2 targeting relationship validated by dual-luciferase reporter assays. BMSCs transfected with miR-143-3p mimic or inhibitor were used to assess exosomes effects on autophagy and podocin expression. In vivo, DKD mice received tail vein injections of modified BMSC-Exos, followed by evaluation of physiological parameters, biochemical indices, and renal histopathology. **Results**: BMSC-Exos were successfully isolated and characterized. Fluorescence microscopy confirmed exosomes internalization by HG-treated MPC5 cells. BMSC-Exos upregulated Beclin1 and LC3-II while downregulating Bcl-2 and desmin, indicating enhanced autophagy. High-throughput sequencing revealed miR-143-3p enrichment in BMSC-Exos, and Bcl-2 was confirmed as a direct target of miR-143-3p. Exosomes from miR-143-3p mimic-transfected BMSCs further promoted autophagy and podocin expression. In DKD mice, BMSC-Exos reduced blood glucose, urinary albumin-to-creatinine ratio (UACR), and ameliorated renal damage, whereas miR-143-3p inhibition attenuated these effects. **Conclusions**: BMSC-Exos deliver miR-143-3p to target Bcl-2, thereby activating Beclin1-mediated autophagy and ameliorating DKD. This study elucidates a novel autophagy regulatory mechanism supporting BMSC-Exos as a cell-free therapy for DKD.

## 1. Introduction

Diabetic kidney disease (DKD), a form of chronic kidney injury induced by DM, affects approximately 40% of patients with type 2 diabetes mellitus (T2DM) and 30% of those with type 1 diabetes mellitus (T1DM). DKD not only increases the risk of renal dysfunction but also significantly raises the incidence of cardiovascular complications, making it a leading cause of end-stage renal disease (ESRD) [1,2]. DM is a chronic endocrine disorder that poses a serious threat to global health. According to recent epidemiological data, approximately 537 million people worldwide are living with diabetes (https://diabetesatlas.org/ accessed on 4 January 2025.), and this number is projected to rise to 693 million by 2045 [3]. Consequently, the global burden of DKD is expected to escalate alongside the increasing prevalence of diabetes.

The pathogenesis of DKD is multifactorial and complex, involving endothelial dysfunction, glomerular hyperfiltration, hemodynamic alterations, and chronic inflammation. Clinically, DKD manifests primarily as proteinuria and progressive loss of renal function, including tubular injury, interstitial fibrosis, and glomerulosclerosis [4]. Currently, therapeutic strategies recommended by clinical guidelines for DKD include renin–angiotensin–aldosterone system inhibitors (RAASi) and sodium-glucose cotransporter 2 (SGLT-2) inhibitors. However, these agents may increase the risk of ESRD and acute kidney injury and thus are not universally suitable for DKD management [5,6]. Therefore, the development of more effective therapeutic strategies requires a deeper understanding of the disease mechanisms and identification of novel therapeutic targets.

Podocytes are terminally differentiated epithelial cells whose foot processes form an integral part of the glomerular filtration barrier (GFB). As podocyte injury represents a crucial pathological event that initiates proteinuria and glomerulosclerosis, thereby facilitating the progression of DKD, therapeutic strategies targeting podocyte dysfunction have attracted increasing attention [7]. Podocyte dysfunction has thus become a key therapeutic target. Emerging evidence suggests that various pathophysiological processes, including impaired autophagy, oxidative stress, immune dysregulation, inflammation, and accumulation of advanced glycation end products (AGEs), are involved in DKD progression [8,9,10,11].

Autophagy is a highly conserved catabolic process that degrades excess or damaged organelles and protein aggregates via the lysosomal pathway [12]. This mechanism maintains cellular homeostasis by removing misfolded proteins, defective organelles, and invasive pathogens [13]. Both autophagy and apoptosis are essential for development and cellular equilibrium. Notably, podocytes exhibit a higher basal level of autophagy compared to other renal intrinsic cells, which is critical for maintaining their physiological function [14]. Multiple autophagy-related genes (ATGs) regulate this process, among which Beclin1 is the first identified autophagy protein in mammals and a key initiator of autophagosome formation [15]. Dysregulation of podocyte autophagy may therefore represent a fundamental mechanism in the onset and progression of DKD.

Mesenchymal stem cells (MSCs) and their secreted exosomes (MSC-Exos) have gained significant attention in regenerative medicine for their potential to alleviate inflammation-related diseases, including DM and DKD. Exosomes, the smallest class of extracellular vesicles (EVs), range in diameter from 30 to 150 nm and mediate intercellular communication by transporting various RNAs and proteins [16]. Compared to MSC-based therapies, which carry risks of tumorigenesis and immune rejection, MSC- exos offer more stable biological characteristics and lower immunogenicity, positioning them as a promising core vehicle for emerging cell-free therapeutic strategies [17].

Importantly, the therapeutic effects of MSC-Exos are closely associated with their cargo of microRNAs (miRNAs), a group of non-coding RNAs that modulate gene expression and remodel the local microenvironment [18,19]. Previous studies have demonstrated the renoprotective potential of MSC-Exos and their miRNA contents in DKD models, although the underlying mechanisms remain to be fully elucidated [20].

Based on this background, the present study utilized bone marrow-derived MSC exosomes (BMSC-Exos) from mice to evaluate their effects on high glucose-induced podocyte injury in vitro and in a mouse model of DKD in vivo. Our findings provide novel insights into the miRNA-mediated therapeutic potential of MSC-Exos in DKD and lay the groundwork for future clinical applications of cell-free therapies.

## 2. Materials and Methods

### 2.1. Cell Culture and Treatment

BMSCs were procured from Zhong Qiao Xin Zhou Biotechnology Co., Ltd. (Shanghai, China; Cat# PRI-MOU-00078). Mouse podocyte clone 5 (MPC5) cells were obtained from Fuheng Biotechnology (Shanghai, China; Cat# FH1009). All cells were maintained in their respective media, DMEM/F12 (Gibco, Thermo Fisher Scientific, Waltham, MA, USA) for BMSCs and DMEM (Gibco, Thermo Fisher Scientific, Waltham, MA, USA) for MPC5 cells, supplemented with 10% fetal bovine serum (FBS; BDbio, Beijing, China; Cat# F802) and 1% penicillin-streptomycin (BKM Bioscience, Shenzhen, China), under standard culture conditions (37 °C, 5% CO_2_). To establish an in vitro DKD model, we have specified that MPC5 cells were cultured at a density of 0.5 × 10^6^ cells per well in a 6-well cell culture plate. MPC5 cells were exposed to high glucose (HG; 50 mM D-glucose) for 24 h, with normal glucose (NG; 5.5 mM D-glucose) serving as the control. After this, we intervened by treating the cells with 50 μg/mL BMSC-Exos or BMSC-Exos treated with miR-143-3p inhibitor [21,22].

### 2.2. Animal Model

Animal experiments were conducted in accordance with the guidelines of the Animal Ethics Committee of The First Affiliated Hospital of Nanchang University (CDYFY-IACUC-202401QR003). Male C57BL/6J mice (6 weeks old) from Tianqin Biotechnology Co., Ltd. (Changsha, China) were housed in a specific pathogen-free (SPF) facility on a 12 h light/dark cycle. After one week of acclimatization with standard chow and ad libitum water access, DKD was induced in two stages. First, mice were fed a high-fat diet (HFD; 60% kcal from fat) for 8 weeks, with controls receiving intraperitoneal (i.p.) PBS injections. Next, streptozotocin (STZ; 30 mg/kg body weight in 0.01 M citrate buffer, pH 4.5; Cat#S0130, Sigma Aldrich, Taufkirchen, Germany) was administered i.p. every other day for a total of three times. One week after the final injection, random tail vein blood glucose levels were measured. Mice with fasting glucose > 11.1 mmol/L were confirmed as diabetic and maintained on HFD for an additional 8 weeks. Urinary microalbumin (MALB) and creatinine (Cr) levels were assessed, and the urinary albumin-to-creatinine ratio (UACR) calculated; those with UACR > 30 mg/g were included in the study [23].

Using a random number table, mice were assigned to four groups (*n* = 6 each): Normal Control, DKD + PBS, DKD + BMSC-Exos-NC, and DKD + BMSC-Exos-miR-143-3p inhibitor. The Normal Control group received standard chow, while others continued on HFD. Weekly tail vein injections included 100 μg of BMSC-Exos in 200 μL 0.9% NaCl, BMSC-Exos transfected with miR-143-3p inhibitor, or an equal volume of 0.9% saline for controls. After 4 weeks, urine samples were collected for MALB and Cr measurement to calculate UACR. Mice were then euthanized, with left kidneys used for histopathological analysis and right kidneys for TEM analysis.

### 2.3. Isolation and Identification of BMSC-Exos

BMSC-Exos were isolated by culturing BMSCs in exosome-depleted FBS (SBI, USA) for 48 h. Conditioned medium was collected and processed sequentially through differential centrifugation steps: 300× *g* for 5 min, 2000× *g* for 20 min, and 10,000× *g* for 30 min, followed by filtration through a 0.22 μm membrane. The supernatant was then ultracentrifuged at 120,000× *g* for 70 min at 4 °C. The resulting pellet was washed with PBS and centrifuged again under identical conditions (120,000× *g* for 70 min). Purified exosomes were resuspended in 100 μL PBS and stored at −80 °C. Characterization included: transmission electron microscopy (TEM; HT7800, Hitachi, Tokyo, Japan) for morphology; nanoparticle tracking analysis (ZetaVIEW, Nanosight Pro, Malvern, Worcestershire, UK) for size distribution; BCA protein assay for concentration quantification; and surface marker validation via nanoparticle flow cytometry (CD9/CD81) and Western blot (CD9/CD63/TSG101, with Calnexin as negative control).

### 2.4. Gene Transfection

Rab27a siRNA, negative control siRNA (siRNA-NC), miR-143-3p mimic, miR-143-3p inhibitor, and corresponding negative controls were synthesized by Ribobio (Guangzhou, China). Cell transfections were performed using Lipofectamine 2000 (Invitrogen, Thermo Fisher Scientific, Waltham, MA, USA) in Opti-MEM I Reduced Serum Medium (Gibco, Thermo Fisher Scientific, Waltham, MA, USA) according to the manufacturer’s protocol. After 6 h of transfection, the medium was replaced with regular complete medium. Transfected cells were harvested 24–48 h post-transfection for RNA and protein extraction, followed by qRT-PCR and Western blot analyses, respectively.

### 2.5. Exosome Tracking

For in vitro tracking, BMSC-Exos were labeled with PKH67 fluorescent dye (Cat#D0031, Solarbio, Beijing, China;), then purified via ultracentrifugation (XPN-100, Beckman Coulter, Brea, CA, USA). MPC5 cells were incubated with PKH67-labeled BMSC-Exos for 24 h, and cellular uptake was visualized using fluorescence microscopy (Olympus IX73, Olympus Corporation, Tokyo, Japan).

### 2.6. Western Blot Analysis

Total protein was lysed using buffer (BKMbio, Shenzhen, China), quantified via BCA assay (Cat# KTD3001, Abbkine, Wuhan, China), separated by SDS-PAGE, and transferred onto PVDF membranes. After blocking with 5% non-fat milk for 2 h, membranes were incubated with primary antibodies at 4 °C overnight followed by HRP-conjugated secondary antibodies(Cat#BA1054, BOSTER, Wuhan, China) (1 h, RT). Protein bands were visualized using ECL substrate (Cat#SQ201, Epizyme, Shanghai, China). Relative expression was quantified by gray-value ratios normalized to β-actin.

### 2.7. Quantitative Real-Time PCR (qRT-PCR)

Total RNA or miRNA was extracted from MPC5, BMSCs, or BMSC-Exos using TRIzol (Invitrogen, Thermo Fisher, Waltham, MA, USA). RNA purity and concentration were measured using Nanodrop 2000 (Thermo Fisher Scientific, Waltham, MA, USA). Reverse transcription was performed using Evo M-MLV RT kits (AG11728, Agbio, Changsha, China) for mRNA, and a stem-loop method for miRNA (AG11745, Agbio, Changsha, China). qPCR was conducted using SYBR Green Pro Taq HS (AG11701, Agbio, Changsha, China) on a Bio-Rad real-time PCR system. The relative expression was calculated using the 2^−ΔΔCt^ method with β-actin or U6 as reference genes. Primers were synthesized by Generay (Shanghai, China); miR-143-3p stem-loop primers were from Agbio (Changsha, China). All primer sequences are listed in Supplementary Table S1.

### 2.8. Dual-Luciferase Reporter Assay

The 3′UTR of Bcl-2 was cloned into the psiCHECK2 luciferase reporter vector. Mutation of the predicted miR-143-3p binding site (based on TargetScan) was introduced via overlapping PCR. Wild-type or mutant reporter plasmids were co-transfected into MPC5 cells with miR-143-3p mimic or control miRNA. After 48 h, firefly and Renilla luciferase activities were measured using the Dual-Luciferase Reporter Assay Kit (Cat#KGE3308, KeyGen Biotech, Nanjing, China) on a microplate reader (SpectraMax iD3, Molecular Devices, Shanghai, China).

### 2.9. Renal Histopathology

Mice were euthanized by cervical dislocation and perfused with 0.9% NaCl. Kidneys were fixed in 4% paraformaldehyde for 24 h, dehydrated in graded ethanol, and embedded in paraffin. Sections (5 μm) were deparaffinized and rehydrated for H&E, PAS, and Masson’s trichrome staining. Slides were sealed with neutral resin and scanned using an Olympus VS200 slide scanner (Olympus Corporation, Tokyo, Japan).

### 2.10. Immunohistochemistry (IHC)

For immunohistochemistry, renal paraffin-embedded sections were deparaffinized in xylene and rehydrated through a graded ethanol series. Antigen retrieval was performed using citrate buffer (pH 6.0). After blocking endogenous peroxidase activity, sections were incubated overnight at 4 °C with primary antibodies against Podocin and Desmin (Cat# A68097 or Cat# A86053, Nature Biosciences, Hangzhou, China). On the following day, the sections were developed using a DAB detection kit (Cat#G1212, Servicebio, Wuhan, China), counterstained with hematoxylin, dehydrated, cleared, and mounted with neutral resin. For negative controls, the primary antibody was omitted. All slides were scanned using an Olympus VS200 slide scanner (Olympus Corporation, Tokyo, Japan).

### 2.11. Transmission Electron Microscopy (TEM)

Fresh kidney tissues (1 mm^3^) were rapidly excised and immediately immersed in electron microscopy fixative solution (Cat#G1102, Servicebio, Wuhan, China) at 4 °C. After fixation, the samples were rinsed three times with 0.1 M phosphate buffer (pH 7.4), and then post-fixed in 1% osmium tetroxide (Cat#18456, Ted Pella Inc., Redding, CA, USA) for 2 h. The tissues were dehydrated through a graded ethanol series (30–100%) and acetone, followed by infiltration and embedding with 812 resin (Cat#90529-77-4, SPI, West Chester, PA, USA). Polymerization was carried out at 60 °C for 48 h. Semi-thin sections (1.5 μm) were stained with toluidine blue for localization. Ultrathin sections (60–80 nm) were obtained using an ultramicrotome (Leica UC7, Leica, Wetzlar, Germany) and mounted onto 200-mesh copper grids coated with carbon support film. The sections were sequentially stained with uranyl acetate (Cat#02624-AB, SPI, West Chester, PA, USA) and lead nitrate (Cat#203580, Sigma, St. Louis, MO, USA), then air-dried. Finally, ultrastructural morphology was observed and imaged using a transmission electron microscope (TEM).

### 2.12. Statistical Analysis

Data are presented as mean ± Standard Deviation (SD). Statistical analyses were conducted using GraphPad Prism 10.1.2. Comparisons between two groups were performed using unpaired two-tailed *t*-tests, while one-way ANOVA followed by Tukey’s multiple comparisons test was used for multiple groups. A *p*-value < 0.05 was considered statistically significant. For IHC and IF quantifications, at least 8 randomly selected fields per mouse were analyzed using ImageJ software (version 1.54g). All experiments were repeated at least three times to ensure reproducibility.

## 3. Results

### 3.1. Characteristics of BMSC-Exos

**Figure 1:**
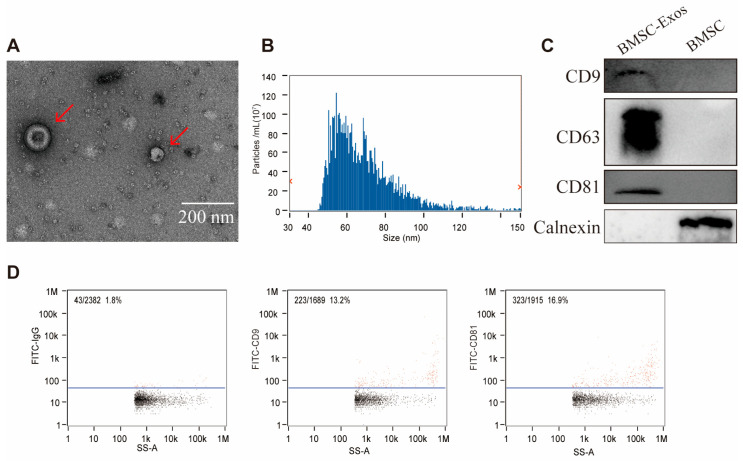
Characterization of BMSC-Exos. (**A**) TEM revealed that BMSC-Exos exhibit the typical cup-shaped morphology; (**B**) NTA showed the particle size distribution of Exos ranging from 30 to 150 nm; (**C**) Western blot analysis confirmed the presence of the exosomal markers CD9, CD63, and TSG101, while the negative marker Calnexin was absent; (**D**) Nano-flow cytometry detected the expression of CD9 and CD81. (Scale bar = 200 nm) All the data were presented as the mean ± SD from 3 biological replicates independent experiments. The red arrow points to BMSC-Exos. The blue line represents the threshold line in the nano-flow cytometry analysis.

BMSC-Exos were characterized using transmission electron microscopy (TEM), nanoparticle tracking analysis (NTA), flow cytometry, and Western blotting. TEM images revealed typical cup-shaped, double-membrane vesicles (Figure 1A), while NTA indicated a size distribution of 30–150 nm with a mean diameter of 68.42 nm (Figure 1B). Flow cytometry and Western blotting confirmed the expression of exosomes markers (CD9/CD63/TSG101) and absence of calnexin (Figure 1C,D), validating successful isolation.

### 3.2. BMSC-Exos Attenuate HG-Induced Podocyte Injury by Restoring Autophagy Activity

**Figure 2:**
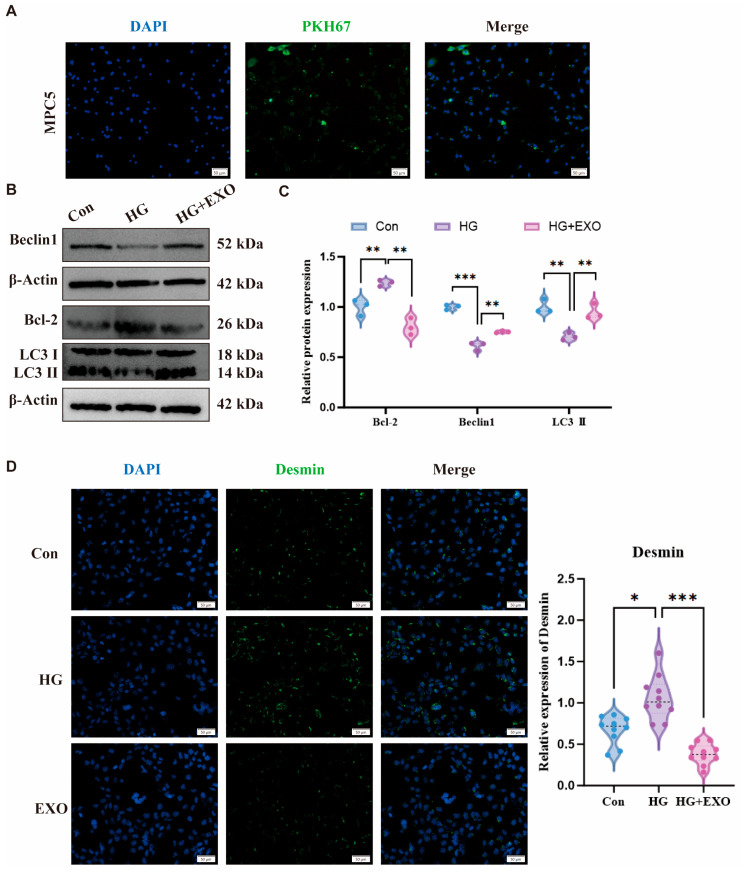
BMSC-Exos are internalized by HG-injured podocytes, activate autophagy, and attenuate cellular injury. (**A**) After 48 h of co-incubation with PKH67-labeled BMSC-Exos, green fluorescence was observed in the cytoplasm of HG-treated MPC5 cells under fluorescence microscopy. (green: PKH67-labeled BMSC-Exos; blue: DAPI-stained nucleus of MPC5 cells). (**B**,**C**) Western blot analysis and relative protein expression of Beclin1, Bcl-2, and LC3 protein expression. (**D**) Immunofluorescence staining of the podocyte injury marker Desmin. All the data were presented as the mean ± SD from 3 biological replicates independent experiments, * *p* < 0.05, ** *p* < 0.01, *** *p* < 0.001 vs. HG.

As shown in Figure 2A, PKH67-labeled BMSC-Exos were co-cultured with high glucose (HG)-treated podocytes for 48 h to assess cellular internalization efficiency. Fluorescence microscopy confirmed cytoplasmic localization of exosomes. Subsequent functional analyses compared Control (normal glucose), HG (50 mM glucose), and HG+Exo (HG + 50 μg/mL BMSC-Exos) groups. Western blot demonstrated that BMSC-Exos upregulated Beclin1 expression, reduced Bcl-2 levels, and enhanced LC3-I/II conversion as shown in Figure 2B,C, indicating autophagy activation. Immunofluorescence further revealed significantly decreased Desmin (podocyte injury marker) in HG+Exo versus HG groups (Figure 2C,D), collectively establishing that BMSC-Exos mitigate HG-induced podocyte damage by activating autophagy via Bcl-2/Beclin1 modulation.

### 3.3. Exosomes miR-143-3p from BMSCs Enhances Autophagy by Targeting the Bcl-2/Beclin1 Axis

**Figure 3:**
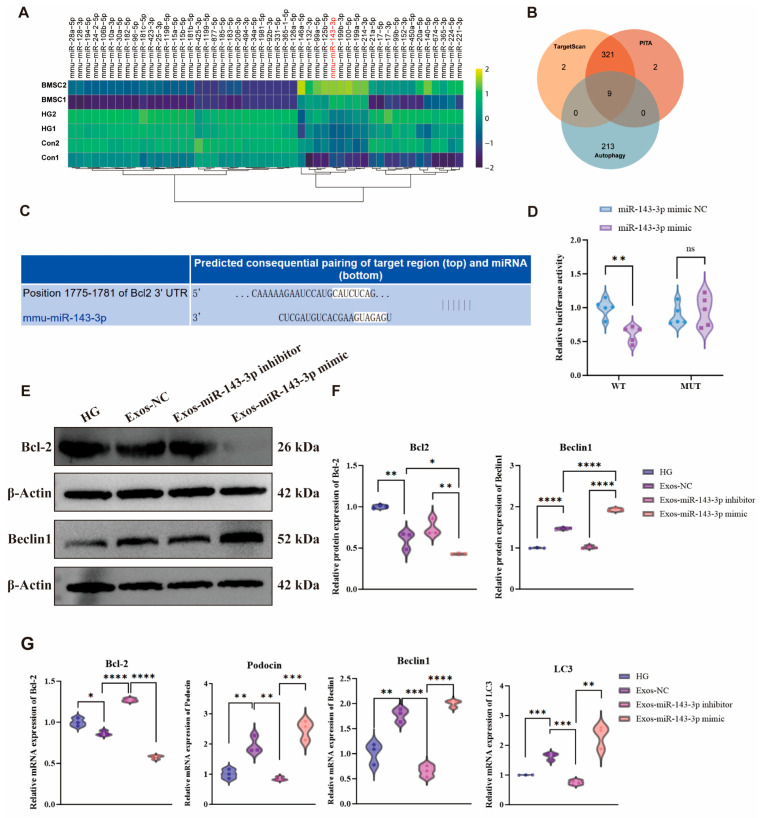
miR-143-3p targets Bcl-2 to regulate autophagy in MPC5 cells. (**A**) High-throughput sequencing analysis revealed significantly higher expression of miR-143-3p in BMSC-Exos compared with podocyte-derived Exos and HG-treated podocyte-derived Exos (Red font: Represents the miR-132-3p gene). (**B**) Online bioinformatic prediction suggested that miR-143-3p potentially targets autophagy-related genes. (**C**) The TargetScan database predicted the binding sites of miR-143-3p on the 3′UTR of Bcl-2. (**D**) Dual-luciferase reporter assay confirmed the direct binding of miR-143-3p to the Bcl-2 3′UTR, resulting in its translational repression. (**E**,**F**) WB analysis of Bcl-2 and Beclin1 expression in HG-induced MPC5 cells treated with Exos derived from BMSCs transfected with miR-143-3p mimics or inhibitors (**E**), along with densitometric quantification (**F**). (**G**) qPCR was used to assess the mRNA expression levels of Bcl-2, Beclin1, LC3, and Podocin in each group. All the data were presented as the mean ± SD from 3 biological replicates independent experiments, * *p* < 0.05, ** *p* < 0.01, *** *p* < 0.001, **** *p* < 0.0001, ns, no significance).

High-throughput sequencing analysis of miRNA expression profiles demonstrated significant upregulation of miR-143-3p in BMSC-derived exosomes compared to podocyte-derived exosomes under both normal and HG conditions (Figure 3A). Bioinformatics prediction using multiple databases identified Bcl-2 as a prime autophagy-related target through Venn diagram intersection analysis (Figure 3B, Supplementary Table S2). Computational modeling further revealed a conserved miR-143-3p binding site within the Bcl-2 3′UTR (Figure 3C). Subsequent dual-luciferase reporter assays provided functional validation, demonstrating that miR-143-3p mimic specifically suppressed wild-type Bcl-2 3′UTR activity but showed no significant effect on mutant constructs in MPC5 cells (Figure 3D). Functional investigation employed BMSCs transfected with miR-143-3p mimic or inhibitor to generate modified exosomes. When administered to HG-treated MPC5 cells, exosomes carrying miR-143-3p mimic significantly downregulated Bcl-2 expression while concurrently enhancing Beclin1 levels and LC3-I to LC3-II conversion, indicating robust autophagy activation. Conversely, exosomes containing miR-143-3p inhibitor increased Bcl-2 while suppressing Beclin1 and LC3-II expression (Figure 3E–G). Notably, podocyte integrity marker Podocin expression showed corresponding upregulation in mimic-treated cells and downregulation with inhibitor treatment (Figure 3G). These findings demonstrate that BMSC-derived exosomes miR-143-3p targets the Bcl-2/Beclin1 axis to enhance podocyte autophagy and mitigate high-glucose induced injury.

### 3.4. BMSC-Derived Exosomes Attenuate DKD Progression In Vivo by Activating Podocyte Autophagy Through miR-143-3p Delivery

**Figure 4:**
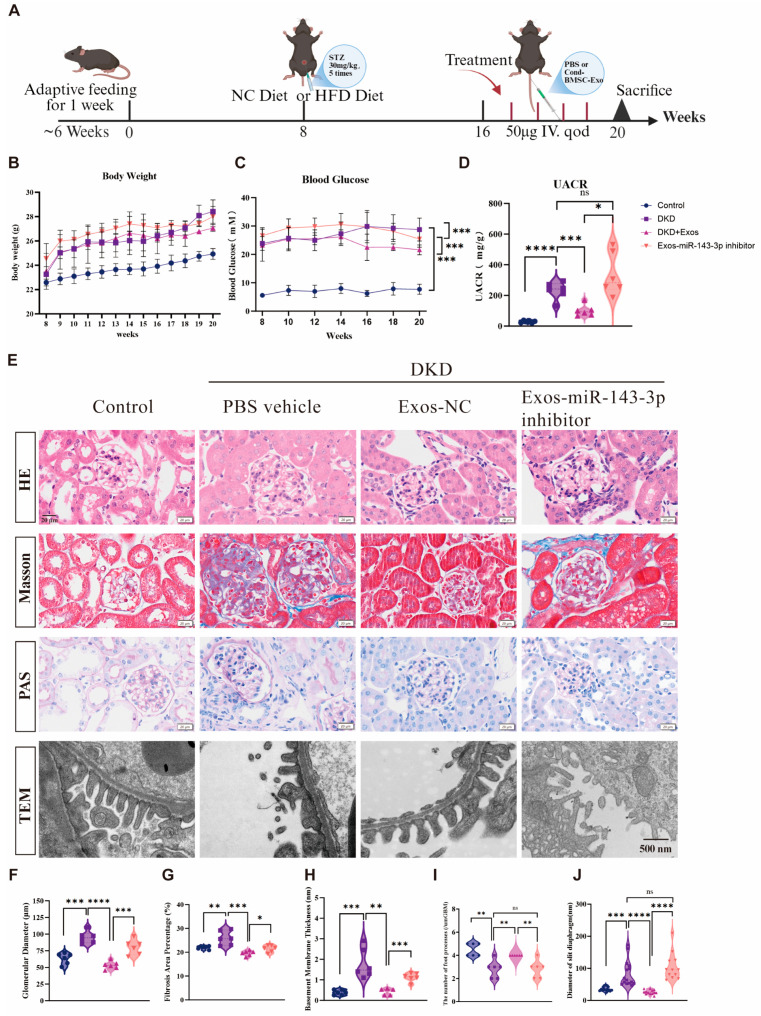
BMSC-derived Exos carrying miR-143-3p attenuate DKD progression and ameliorate renal structural damage in vivo. (**A**) Schematic diagram of the in vivo experimental protocol. (**B**) Body weight changes in mice from each group during the intervention period. (**C**) Blood glucose levels in each group during the intervention. (**D**) Urinary albumin-to-creatinine ratio (UACR) in each group after treatment. (**E**) Representative images of renal tissues stained with H&E, Masson, and PAS, as well as TEM observation of glomerular podocyte ultrastructure in each group; (**F**) Quantification of glomerular diameter (μm); (**G**) Fibrotic area percentage; (**H**) Thickness of the glomerular basement membrane; (**I**) Number of podocyte foot processes per unit length (μm); (**J**) Width of the slit diaphragm (nm). All data are presented as the mean ± SD from 6 independent biological replicates (*n* = 6). Statistical significance was determined using one-way ANOVA, * *p* < 0.05, ** *p* < 0.01, *** *p* < 0.001, **** *p* < 0.0001, ns, no significance). (Figure 4E Scale bar of the first three rows = 20 μm, Scale bar of the last row = 500 nm).

**Figure 5:**
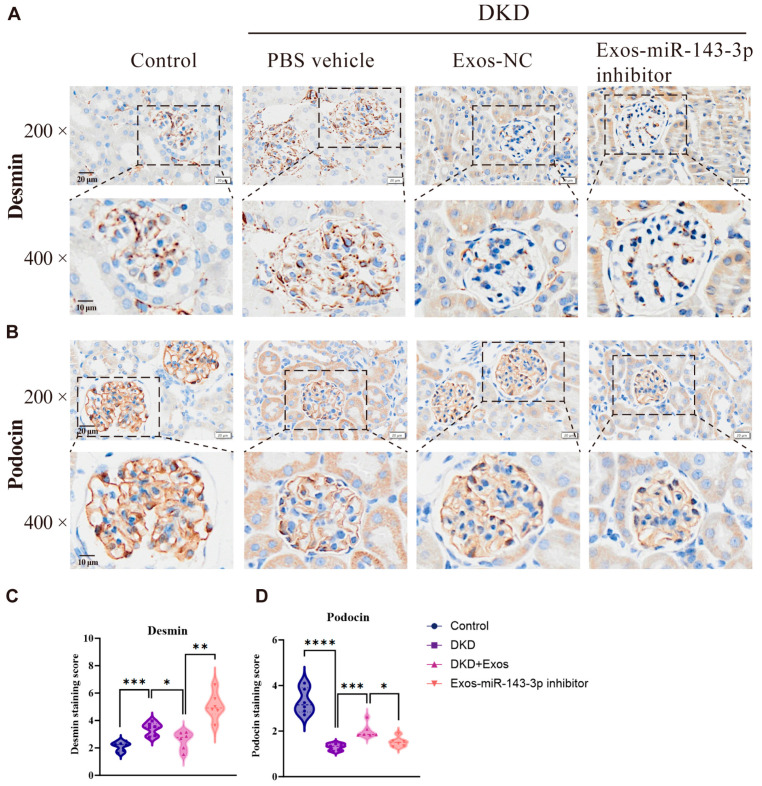
Immunohistochemical detection of Podocin and Desmin expression levels in renal tissues of mice from different groups. (**A**,**C**) IHC staining results of Desmin, a marker of podocyte injury, in glomeruli (**A**), and semi-quantitative analysis of the positive staining area (**C**); (**B**,**D**) IHC staining results of Podocin, a podocyte-specific marker, (**B**), and semi-quantitative analysis of the positive staining area (**D**). All data are presented as the mean ± SD from 6 independent biological replicates (*n* = 6). Statistical significance was determined using one-way ANOVA * *p* < 0.05, ** *p* < 0.01, *** *p* < 0.001, **** *p* < 0.0001. (Figure 5A Scale bar of the first row = 20 μm, Scale bar of the second row = 10 μm, Figure 5B Scale bar of the first row = 20 μm, Scale bar of the second row = 10 μm).

Building upon in vitro evidence that BMSC-derived exosomes mitigate podocyte injury via miR-143-3p-mediated targeting of the Bcl-2/Beclin1 autophagy pathway, we established a DKD mouse model to evaluate therapeutic efficacy. The Animals were grouped according to Materials and Methods. The experimental protocol is presented in Figure 4A. Compared to Normal Control, DKD mice exhibited significant hyperglycemia, increased body weight, and elevated UACR. Exos-NC intervention substantially reduced blood glucose and UACR relative to PBS-treated DKD controls, while Exos-miR-143-3p inhibitor aggravated both parameters (Figure 4B–D). Pathological assessment revealed that Exos-NC ameliorated glomerular hypertrophy, inflammatory infiltration, and collagen deposition observed in H&E, Masson, and PAS staining, whereas Exos-miR-143-3p inhibitor failed to improve these lesions. Ultrastructural analysis via TEM confirmed preserved podocyte foot processes and slit diaphragms in Exos-NC group, contrasting with extensive effacement in DKD controls and inhibitor groups (Figure 4E–J). As depicted in Figure 5A–D, immunohistochemical analysis further demonstrated upregulated podocin and downregulated desmin expression with Exos-NC treatment, indicating enhanced podocyte integrity, while miR-143-3p inhibition produced opposite effects. These integrated physiological, biochemical, histological, and molecular findings collectively validate that exosomes miR-143-3p attenuates DKD progression through autophagy activation and podocyte protection.

## 4. Discussion

DKD is a leading cause of diabetes-related mortality [24,25], characterized by glomerular injury and progressive renal dysfunction [26,27]. MSC-Exos have emerged as promising therapeutic agents due to their ability to deliver bioactive molecules [28,29], particularly miRNAs, to modulate inflammation, fibrosis, and apoptosis [30]. For example, Wang et al. [31] reported that MSC-Exos alleviate DKD via regulation of the NLRP3 signaling pathway, and Cui et al. [22] showed that MSC-Exos reduce apoptosis and epithelial–mesenchymal transition through miR-424-5p. Among these, miR-143-3p has been implicated in kidney protection. Autophagy dysfunction in podocytes contributes to DKD progression [12,32], with the Bcl-2/Beclin1 axis playing a pivotal regulatory role [32,33]. In many experimental DKD models (including STZ-induced diabetes), STZ causes β-cell loss and persistent hyperglycaemia that promotes oxidative stress, ER stress, and inflammatory signaling in the kidney; these stressors impair autophagic flux in podocytes (for example via reduced LC3-II and Beclin-1 activity and altered expression of ATG proteins), thereby accelerating podocyte injury, apoptosis, and glomerulosclerosis [34]. Restoring podocyte autophagy—whether pharmacologically or biologically—has been shown to reduce podocyte apoptosis, preserve cytoskeletal and slit-diaphragm integrity, and attenuate progression of glomerular lesions in diabetic models [2]. Therefore, in this study we investigated whether bone marrow MSC–derived exosomal miR-143-3p mitigates podocyte injury in DKD by targeting the Bcl-2/Beclin1 pathway to restore autophagy, a mechanism that could counteract STZ-induced autophagy impairment and protect glomerular function.

Obtaining high-quality exosomes is critical to experimental reliability. We successfully isolated BMSC-Exos using ultracentrifugation and characterized them via TEM, NTA, and Western blot analysis, meeting the latest criteria of the Minimal Information for Studies of Extracellular Vesicles (MISEV2023) and the features described by Raghu Kalluri et al. [35,36]. Although we did not conduct specific tests on exosome storage and biological activity in this study, prior research has provided valuable insights into the stability and bioactivity of exosomes under various storage conditions. Studies have demonstrated that exosomes stored at temperatures below −70 °C exhibit superior stability, preserving key biomarkers such as ALIX, HSP70, and TSG101, which are crucial for their functionality [37,38]. In contrast, storage at room temperature or repeated freeze–thaw cycles can result in significant degradation of exosome components, including proteins and RNA [37]. To ensure the long-term stability and bioactivity of exosomes in our study, we stored them at −80 °C immediately after isolation. Our NTA analysis further confirmed that exosome particle count remained stable even after 2 months of storage at this temperature. This practice, supported by previous studies, ensures that exosomes retain their biological activity during long-term storage and repeated injection. In vitro, we modeled DKD by exposing MPC5 cells to HG, a well-established approach to mimic podocyte injury observed in DKD [39,40]. Our results confirmed that BMSC-Exos are efficiently internalized by MPC5 cells under HG conditions and significantly reverse HG-induced autophagy dysfunction and cellular injury, evidenced by upregulation of Beclin1 and LC3-II and downregulation of Bcl-2. Bcl-2 is known to inhibit autophagy through its interaction with Beclin1, and this suppression can be lifted when Bcl-2 is inactivated [41]. Thus, BMSC-Exos may exert protective effects on podocytes by modulating the autophagy pathway to alleviate HG-induced damage.

Mechanistically, our findings establish that BMSC-derived exosomes deliver miR-143-3p to podocytes, where it directly targets Bcl-2 via 3′-UTR binding, thereby relieving Bcl-2-mediated suppression of Beclin1 and initiating autophagic flux, a sequential regulatory cascade confirmed through miRNA gain/loss-of-function experiments. Beyond restoring autophagy homeostasis, this miR-143-3p-mediated mechanism significantly upregulates podocin expression, demonstrating concurrent restoration of podocyte structural integrity and slit diaphragm function. These results delineate a novel miR-143-3p/Bcl-2/Beclin1 signaling axis that coordinately mitigates high glucose-induced podocyte injury, providing fundamental insights into exosome-mediated cytoprotection in diabetic kidney disease pathogenesis.

In vivo, studies were conducted using only male mice. This decision was based on several factors, including the more stable hyperglycemia observed in male mice, which enhances the consistency and reproducibility of diabetes modeling [42]. Additionally, the hormonal fluctuations in female mice due to estrous cycles can introduce variability in metabolic responses such as insulin sensitivity, which may affect the outcome of diabetes-related studies. Male mice exhibit more stable physiological conditions under metabolic stress, providing a more reliable model for studying DKD progression [42].

Additionally, the inherent targeting ability of MSC-derived exosomes, as demonstrated by previous studies, is highly relevant to the therapeutic potential of BMSC-Exos in our DKD model. For instance, Hu et al. [43] reported that exosomes, following intravenous injection, were widely distributed across various organs, including the liver, lung, and kidneys. Notably, these exosomes accumulated in the obstructed kidney within 24 h post-injection, with maximal accumulation observed at 72 h. Even at 10 days post-injection, exosomes remained detectable in the obstructed kidney, albeit at reduced levels, while only minimal accumulation occurred in the contralateral kidney. These findings suggest a preferential targeting of exosomes to kidney tissues, supporting the hypothesis that BMSC-Exos in our DKD model likely follow a similar distribution pattern. The targeting ability reported in the literature reinforces the potential of BMSC-Exos for kidney-specific therapeutic applications in DKD. Thus, the doses of BMSC-Exos and BMSC-Exos-miR-143-3p inhibitor were selected based on the study by Hu et al.

Our in vivo studies revealed that BMSC-Exos carrying miR-143-3p significantly attenuated disease progression, evidenced by partial reduction in blood glucose levels, decreased UACR, and improved renal histopathology. Most notably, this therapeutic efficacy was strictly dependent on miR-143-3p delivery, as exosomes with inhibited miR-143-3p expression not only failed to confer protection but exacerbated kidney injury, conclusively establishing miR-143-3p as the core effector molecule in MSC-Exos-mediated renoprotection. Histological analyses further confirmed partial restoration of renal architecture and function in BMSC-Exos-treated DKD mice. Collectively, these results align with established mechanisms of miRNA-regulated renal fibrosis and autophagy, thereby reinforcing miR-143-3p’s potential as a promising therapeutic target for DKD. However, the importance of gender-specific effects should be recognized, and we call for future studies to explore female models to ensure more comprehensive research.

To our knowledge, this study establishes the novel mechanism by which BMSC-Exos mitigate DKD progression through miR-143-3p delivery. Specifically, BMSC-Exos transport miR-143-3p to podocytes, where it plays a crucial role in restoring cellular homeostasis that is disrupted by high glucose conditions. In DKD, elevated glucose levels induce a series of stress responses in podocytes, including oxidative stress, inflammation, and impaired autophagy, all of which contribute to podocyte injury and glomerulosclerosis. Autophagy, a critical cellular process for maintaining homeostasis and protecting against stress, is notably suppressed in podocytes under high glucose conditions. This suppression is associated with the inhibition of key autophagic proteins, such as Beclin1, which is essential for the initiation of autophagy, and LC3, which regulates autophagic vesicle formation. In our study, BMSC-Exos deliver miR-143-3p to podocytes, where it specifically targets Bcl-2, a protein that typically inhibits Beclin1’s activation and the initiation of autophagy. By suppressing Bcl-2 expression, miR-143-3p enhances the activation of Beclin1, thereby restoring autophagic flux and allowing the podocytes to effectively manage the increased cellular stress induced by hyperglycemia. This restoration of autophagy reduces podocyte apoptosis, improves cellular function, and protects against glomerular damage, which are crucial factors in preventing the progression of DKD. Compared to traditional MSC therapies, which often require direct cell transplantation and may face challenges such as immune rejection and limited retention at the target site, BMSC-Exos offer a more efficient and targeted approach. The exosomes, being nanosized vesicles, are capable of targeting specific cells, such as podocytes, and delivering their cargo directly to the site of injury. This targeted delivery, coupled with the low immunogenicity of exosomes, significantly reduces the risk of adverse immune responses [44]. Furthermore, exosomes have enhanced stability in circulation, allowing for prolonged therapeutic effects. These advantages position exosome-based strategies as a promising alternative to traditional MSC therapies, offering more precise, effective, and safer treatments for DKD.

Despite robust in vitro and in vivo evidence supporting the central role of miR-143-3p, the therapeutic effects of BMSC-Exos likely involve synergistic contributions from co-packaged miRNAs and protein cargo. Comprehensive characterization of exosomes components through multi-omics approaches is needed to delineate these cooperative mechanisms. Furthermore, while our data confirm that autophagy restoration mediates podocyte protection, the downstream molecular events, particularly temporal flux dynamics and substrate-specific degradation pathways, require deeper mechanistic dissection. Although the murine model replicates key pathological features of human DKD, inherent interspecies differences in disease progression necessitate validation in human-relevant systems. Critical next steps include: optimizing scalable exosome production and targeted delivery platforms; establishing patient-derived podocyte models to verify therapeutic efficacy; and conducting preclinical safety/toxicity profiling prior to clinical translation.

## 5. Conclusions

**Figure 6:**
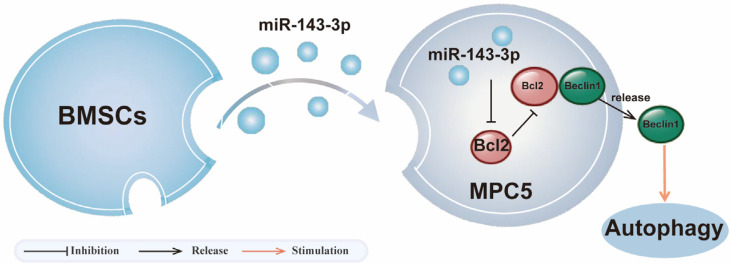
Schematic of BMSC-Exos miR-143-3p-mediated protection against podocyte injury in DKD.

In summary, our study reveals that BMSC-Exos alleviate DKD progression by delivering miR-143-3p, which targets the Bcl-2/Beclin1 pathway to restore autophagy in HG-injured podocytes (Figure 6). This mechanism protects podocytes from hyperglycemia-induced damage and offers a promising therapeutic strategy for DKD. These findings provide theoretical and experimental support for the development of exosome-based, cell-free therapies and offer a novel direction for the precision treatment of DKD.

## Data Availability

The raw sequencing data have been deposited in the NCBI Sequence Read Archive (SRA) under the BioProject ID PRJNA1301607 and will be available upon publication. Other data that support the findings of this study are available from the corresponding author upon reasonable request.

## Supplementary Materials

The following supporting information can be downloaded at: https://www.mdpi.com/article/10.3390/biomedicines14010184/s1, Table S1: Primers for qPCR; Table S2: Target Gene Prediction Table.

## Abbreviations

The following abbreviations are used in this manuscript:

DKD	diabetic kidney disease
DM	diabetes mellitus
BMSC-Exos	bone marrow mesenchymal stem cell-derived exosomes
HG	high glucose
MPC5	mouse podocyte clone 5
Bcl-2	B-cell lymphoma 2
LC3	microtubule-associated protein 1 light chain 3
IF	immunofluorescence
UACR	urinary albumin-to-creatinine ratio
SD	standard deviation
T2DM	type 2 diabetes mellitus
T1DM	type 1 diabetes mellitus
ESRD	end-stage renal disease
RAASi	renin–angiotensin–aldosterone system inhibitor
SGLT-2	sodium-glucose cotransporter 2
GFB	glomerular filtration barrier
AGEs	advanced glycation end products
ATGs	autophagy-related genes
MSCs	mesenchymal stem cells
MSC-Exos	mesenchymal stem cell-derived exosomes
EVs	extracellular vesicles
miRNAs	microRNAs
HFD	high-fat diet
STZ	streptozotocin
PBS	phosphate-buffered saline
Cr	creatinine
MALB	microalbumin
SPF	specific pathogen-free
DMEM	Dulbecco’s modified Eagle medium
FBS	fetal bovine serum
TEM	transmission electron microscopy
qRT-PCR	quantitative real-time polymerase chain reaction
siRNA	small interfering RNA
PVDF	polyvinylidene difluoride
ECL	enhanced chemiluminescence
UTR	untranslated region
DAB	diaminobenzidine
HRP	horseradish peroxidase
BCA	bicinchoninic acid
