# Supplementary material for: Mesenchymal Stem Cell-Derived Exosomes miR-143-3p Attenuates Diabetic Kidney Disease by Enhancing Podocyte Autophagy via Bcl-2/Beclin1 Pathway
Source: Biomedicines. 2026 Jan 14;14(1):184. doi: 10.3390/biomedicines14010184 (PMC12838913; doi:10.3390/biomedicines14010184)
Supplement: Supplementary file 1 [file biomedicines-14-00184-s001.zip › Supplementary Table S1.pdf]

Table.S1

Primers for qPCR:

Gene	Forward（5’-3’）	Reverse（5’-3’）	Product(bp)
LC3	TTCGGGTIGCTCTTTTGGGT	GACAGGCAAGGGCCTAACAA	98
Beclin1	TAGCTGAAGACCGGGCGAT	CCACCCAGGCTCGTTCTAC	92
Bc1-2	GCTGGGGATGACTTCTCTCG	CCACAATCCTCCCCCAGTTC	148
U6	TTCGGCAGCACATATACT	AATTTGCGTGTCATCCTT	78
β-actin	GATCAGCAAGCAGGAGTACGA	GGGTGTAAAACGCAGCTCA	92
miR-143-3p	CGCGTGAGATGAAGCACTG	AGTGCAGGGTCCGAGGTATT	

Stem-loop primers for miR-143-3p RT-PCR：

GTCGTATCCAGTGCAGGGTCCGAGGTATTCGCACTGGATACGACGAGCTA
